# In Search of a Phosphorus Dendrimer-Based Carrier of Rose Bengal: Tyramine Linker Limits Fluorescent and Phototoxic Properties of a Photosensitizer

**DOI:** 10.3390/ijms21124456

**Published:** 2020-06-23

**Authors:** Krzysztof Sztandera, Monika Marcinkowska, Michał Gorzkiewicz, Anna Janaszewska, Regis Laurent, Maria Zabłocka, Serge Mignani, Jean Pierre Majoral, Barbara Klajnert-Maculewicz

**Affiliations:** 1 Department of General Biophysics, Faculty of Biology and Environmental Protection, University of Lodz, 141/143 Pomorska St., 90-236 Lodz, Poland; krzysztof.sztandera@unilodz.eu (K.S.); monika.marcinkowska@biol.uni.lodz.pl (M.M.); michal.gorzkiewicz@biol.uni.lodz.pl (M.G.); anna.janaszewska@biol.uni.lodz.pl (A.J.); 2 Laboratoire de Chimie de Coordination, CNRS, 205 Route de Narbonne, BP44099, 31077 Toulouse CEDEX 4, France; regis.laurent@lcc-toulouse.fr; 3 LCC-CNRS, Université de Toulouse, CNRS, 31077 Toulouse, France; 4 Centre of Molecular and Macromolecular Studies, Polish Academy of Sciences, 112 Sienkiewicza St., 90-363 Lodz, Poland; zabloc@cbmm.lodz.pl; 5 CQM-Centro de Quimica da Madeira, Universidade da Madeira, Campus da Penteada, 9020-105 Funchal, Portugal; serge_mignani@orange.fr; 6 Laboratoire de Chimie et de Biochimie Pharmacologiques et Toxicologique, Université Paris Descartes, PRES Sorbonne Paris Cité, CNRS UMR 860, 45 rue des Saints Peres, 75006 Paris, France; 7 Leibniz-Institut für Polymerforschung Dresden e.V., 6 Hohe St., 01069 Dresden, Germany

**Keywords:** skin cancer, photodynamic therapy, phosphorus dendrimers, rose bengal (RB)

## Abstract

Photodynamic therapy (PDT) is a skin cancer treatment alternative to chemotherapy and radiotherapy. This method exploits three elements: a phototoxic compound (photosensitizer), light source and oxygen. Upon irradiation by light of a specific wavelength, the photosensitizer generates reactive oxygen species triggering the cascade of reactions leading to cell death. The positive therapeutic effect of PDT may be limited due to low solubility, low tumor specificity and inefficient cellular uptake of photosensitizers. A promising approach to overcome these obstacles involves the use of nanocarrier systems. The aim of this initial study was to determine the potential of the application of phosphorus dendrimers as carriers of a photosensitizer—rose bengal (RB). The primary goal involved the synthesis and in vitro studies of covalent drug–dendrimer conjugates. Our approach allowed us to obtain RB–dendrimer conjugates with the use of tyramine as an aromatic linker between the carrier and the drug. The compounds were characterized by FT-IR, ^1^H NMR, ^13^C NMR, ^31^P NMR, size and zeta potential measurements and spectrofluorimetric analysis. The dialysis to check the drug release from the conjugate, flow cytometry to specify intracellular uptake, and singlet oxygen generation assay were also applied. Finally, we used MTT assay to determine the biological activity of the tested compounds. The results of our experiments indicate that the conjugation of RB to phosphorus dendrimers via the tyramine linker decreases photodynamic activity of RB.

## 1. Introduction

According to World Health Organization (WHO), the incidence of both non-melanoma and melanoma skin cancers has been increasing over the past decades. Currently, between two and three million non-melanoma skin cancers and 132,000 melanoma skin cancers occur globally each year [1]. Chemotherapy and radiotherapy are used successfully in the treatment of these types of cancer, but cause many complications and side effects [2,3]. Limited specificity of those therapies provokes the search for safer and more effective treatment strategies [4]. Photodynamic therapy (PDT) is a very interesting alternative method of skin cancer therapy [5]. In particular, the effectiveness of PDT depends on three factors: the presence of molecular oxygen, a specific wavelength of light, and the use of special particles called photosensitizers or photosensitizing agents (PSs) [6].

**Figure 1:**
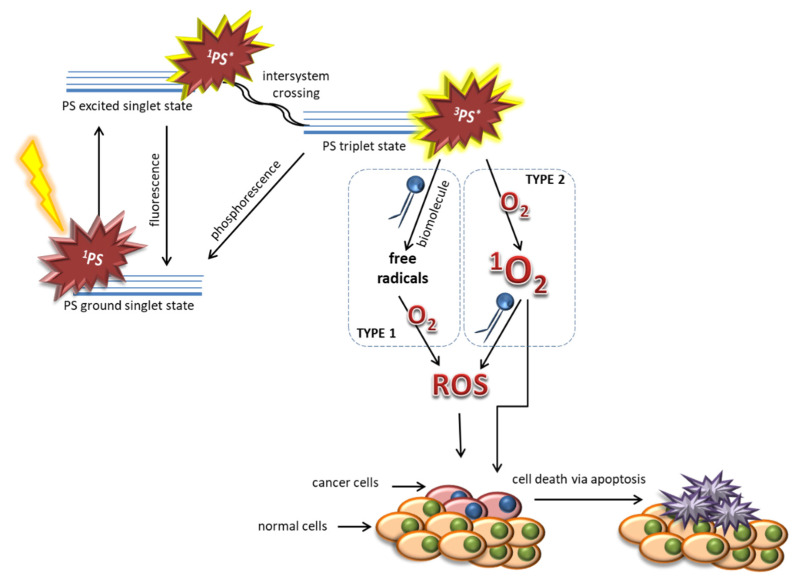
Two possible mechanisms of the photodynamic effect.

In the first step of PDT, photosensitizer absorbs the quantum of light and goes into the singlet excited state. Then, due to the non-radiant intercrossing transition, the PS reaches the excited triplet state. This form is capable of exciting biomolecules around it. The excess of energy is transferred to oxygen which triggers the production of reactive oxygen species (ROS), causing tissue damage. The result of such mechanism involves the accumulation of superoxide anion radicals. However, another mechanism seems to be more significant in photodynamic therapy. After the occurrence of the excited triplet state of the photosensitizer, there is a direct transfer (without participation of biomolecules) of energy to the oxygen, which leads to the formation of singlet oxygen. Singlet oxygen belongs to very strong oxidizers, so its life in tissue is very short. Both mechanisms (Figure 1) take place in photosensitization and they share the resultant outcome of toxicity, which depends on the concentration of oxygen in the tissue, the pH, the dielectric constant of the tissue and other factors [7,8,9]. 

Some recent studies proved that covalent attachment of photosensitizers to nanomaterials can increase phototherapy outcome and reduce their side effects [10,11]. Metal nanoparticles [12], polymeric micelles [13], and ceramic-based nanoparticles [14] were recognized as potential carriers of photosensitizers. Moreover, it was previously demonstrated that the use of nanoparticles avoids the transfer of the photosensitizer to healthy tissues [15,16]. 

Phosphorus dendrimers are very promising carriers, e.g., for therapeutic siRNA [17]. Polycationic phosphorus dendrimers [18] are also able to transport negatively charged molecules, such as rose bengal (RB) [19]. Combining cationic charges present on the surface of phosphorus dendrimers and anionic charges of the RB allows to obtain complexes in which the photosensitizer retains its properties in vitro [20]. However, the complexes can show a very serious disadvantage—low stability in vivo. 

Therefore, in the initial study, we decided to design more stable conjugates containing RB and polycationic phosphorus dendrimers using a linker—a small particle working as a bridge between RB and phosphorus dendrimers.

The aim of our work was to chemically modify rose bengal in order to graft it on the surface of phosphorus dendrimers and to investigate the influence of the resulting conjugates on the effectiveness of phototherapy in vitro.

## 2. Results

### 2.1. Synthesis and Characterization of Conjugates

**Figure 2:**
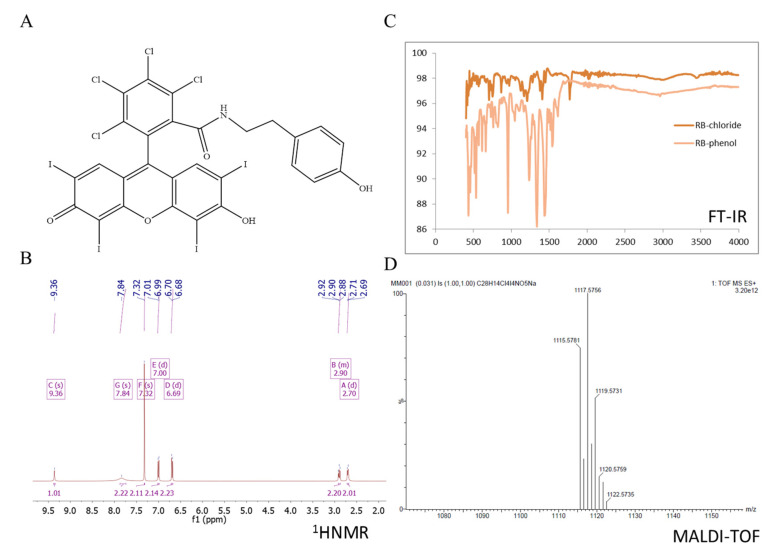
Results for rose bengal–phenol: (**A**) chemical formula; (**B**) ^1^H NMR spectra (DMSO-d6; ppm), (**C**) FT-IR spectra (neat), (**D**) MALDI-TOF spectrum of obtained rose bengal–phenol at *m*/*z* = 1117.57. The calculated molecular weight is M: 1116.

Our approach allowed to obtain rose bengal–phosphorus dendrimer conjugates using tyramine as a linker. The compounds were characterized by FT-IR, ^1^H NMR, ^13^C NMR and ^31^P NMR. Synthesis of rose bengal–phenol (RB-TYR) required a two-step reaction. The first step involves the conversion of the carboxylic group of rose bengal to an acid chloride group which in turn reacts with tyramine, forming an amide bond. The progress of reactions was confirmed by TLC, MALDI-TOF, ^1^H NMR and FT-IR spectroscopy (Figure 2). For example, the reaction was monitored by FT-IR: we observed the disappearance of the band at 1780 cm^−1^ (for acid chloride) on behalf of a new one at 1614 cm^−1^ (for amide group), which confirmed the conversion of acid chloride into the corresponding phenol (Figure 2C). 

The second step of the reaction concerns the partial grafting of such a modified RB derivative (65 µmol) onto the surface of phosphorus dendrimers (21 µmol). For this purpose we prepared phosphorus dendrimers of generation 1 to 3 (G1, G2 and G3) with P(S)Cl_2_-terminal groups according to the synthesis pathway previously described [21]. The third step involved the addition of amino groups on the remaining P-Cl bonds on the outer shell of the phosphorus dendrimers followed by protonation of the grafted amino groups with HCl.

**Figure 3:**
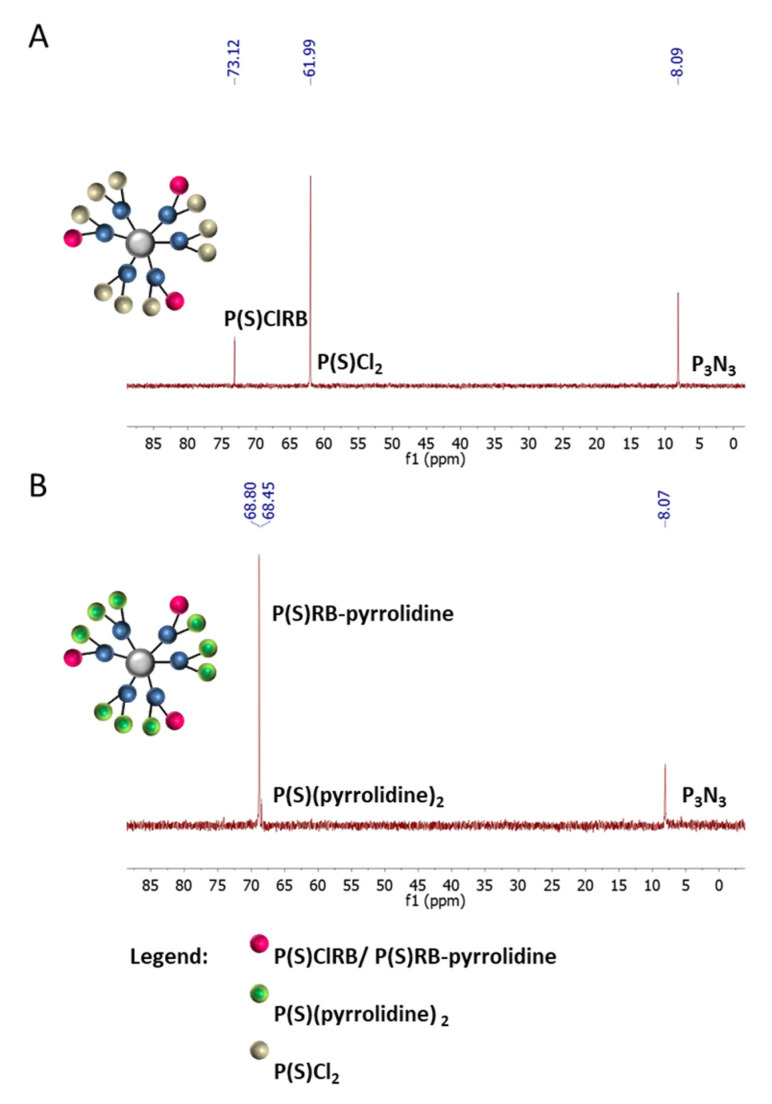
^31^P NMR spectra for: (**A**) G1–3RB (THF-d8; ppm), (**B**) G1–3RB–9 pyrrolidine (G1-3RB-pyrro)(CDCl_3_; ppm).

^31^P NMR spectra for G1–3RB (A) and G1–3RB–9 amine (B) are presented in the Figure 3. On the upper panel (A), we observed the presence of a signal at 73.13 ppm characteristic for the P(S)ClRB group occurring on the surface of modified phosphorus dendrimer and a signal at 61.99 ppm characteristic for unmodified P(S)Cl_2_ groups. The signals at 8.09 ppm (6A) and 8.07 ppm (6B) correspond to the core (P_3_N_3_). On the lower panel (B), after the grafting of amino groups, we observed the disappearance of both chemical shifts (73.13 and 61.99 ppm), on behalf of two new signals at 68.45 ppm (-P(S)RB–pyrrolidine) and 68.80 ppm (-P(S)(pyrrolidine)_2_). ^1^H NMR values were in agreement with the presence of three molecules of RB and nine molecules of pyrrolidine on the surface of the dendrimer.

**Figure 4:**
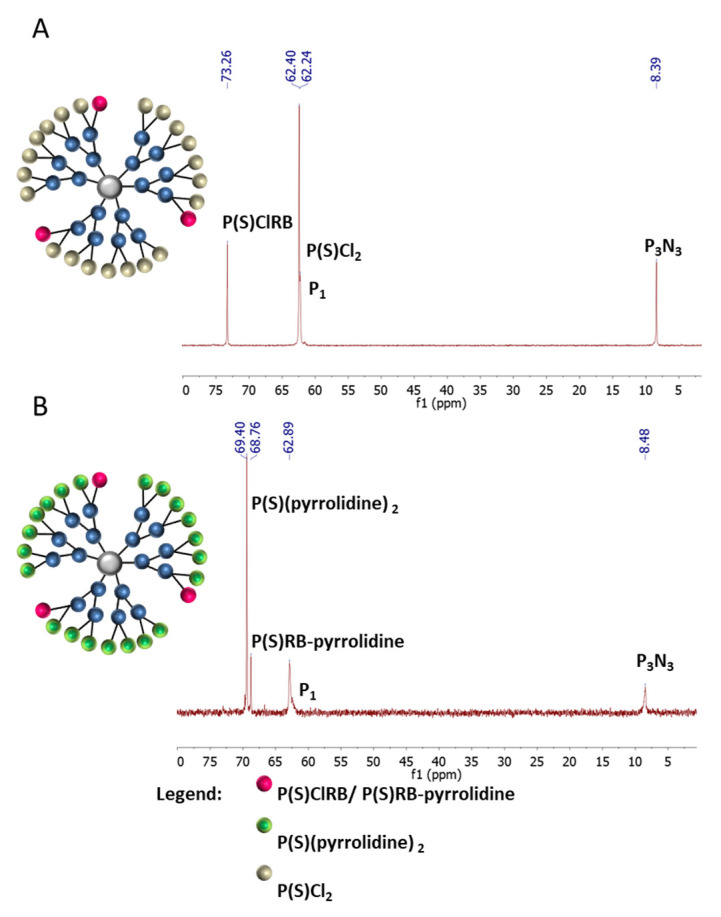
^31^P NMR spectra for: (**A**) G2–3RB (THF-d8; ppm), (**B**) G2–3RB–21 pyrrolidine (G2-3RB-pyrro) (CDCl_3_; ppm).

Using the same technique, we modified the surface of the G2 phosphorus dendrimer containing 12P(S)Cl_2_-terminal groups with 3 equivalents of RB derivative and 21 equivalents of amine. In Figure 4, ^31^P NMR spectra for G2–3RB (A) and G2–3RB–21 amine (B) are presented. On the upper panel (A) we observed a chemical shift at 73.26 ppm characteristic for the P(S)ClRB group occurring on the surface of the modified phosphorus dendrimer and signals at 8.39 (core), 62.40 (-P(S)Cl_2_) and 62.24 (P_1_) ppm. The signal at 8.39 ppm corresponds to the core (P_3_N_3_). On the lower panel (B), after the addition of pyrrolidine, we observed the appearance of new signals at the 68.76 (-P(S)RB–pyrrolidine) and 69.40 ppm (-P(S)(pyrrolidine)_2_). ^1^H NMR values were in agreement with the presence of 3 molecules of RB and 21 molecules of pyrrolidine on the surface of the dendrimer.

**Figure 5:**
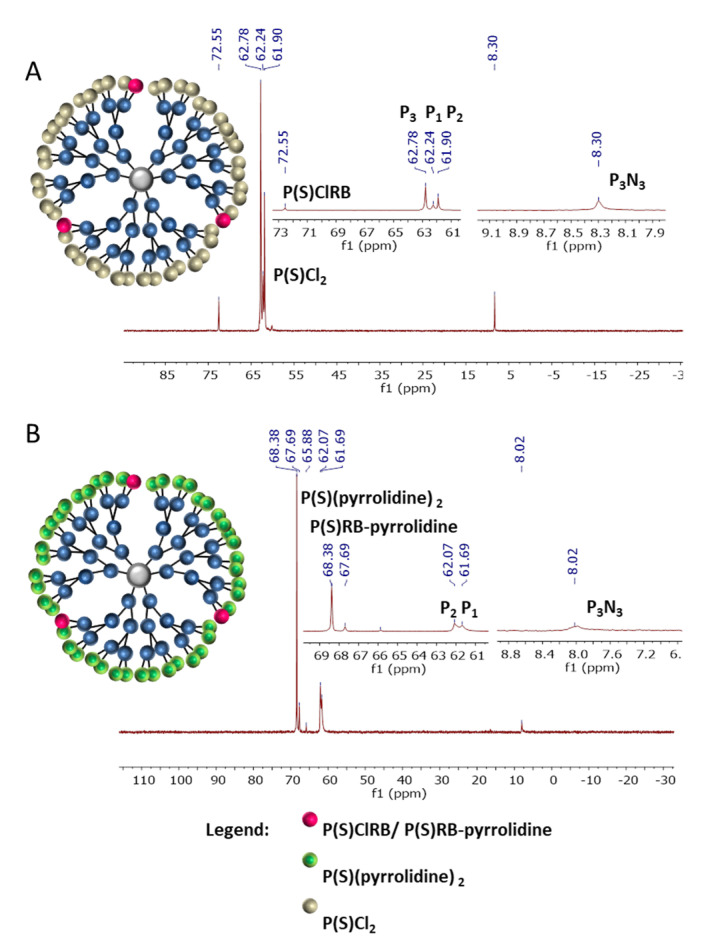
^31^P NMR spectra for: (**A**) G3–3RB (CDCl_3_; ppm); (**B**) G3–3RB–45 pyrrolidine (G3-3RB-pyrro)(CDCl_3_; ppm).

Finally, we modified the surface of the G3 phosphorus dendrimer containing 24 P(S)Cl_2_-terminal groups with three equivalents of RB derivative and 45 equivalents of amine. In Figure 5, ^31^P NMR spectra for G3–3RB (A) and G3–3RB–45 pyrrolidine (B) are presented. On the upper panel (A) we observed new chemical shift at 72.55 ppm characteristic for the P(S)ClRB group occurring on the surface of the modified phosphorus dendrimer and other signals at 8.30 (core), 61.90 (P_2_), 62.24 (P_1_), 62.78 (P_3_) ppm. On the lower panel (B), after the addition of pyrrolidine, we observed the appearance of new signals at 67.69 (-P(S)RB–pyrrolidine) and 68.38 ppm (-P(S)(pyrrolidine)_2_). ^1^H NMR values were in agreement with the presence of 3 molecules of RB and 45 molecules of pyrrolidine on the surface of the dendrimer.

### 2.2. Spectroscopy Studies

**Figure 6:**
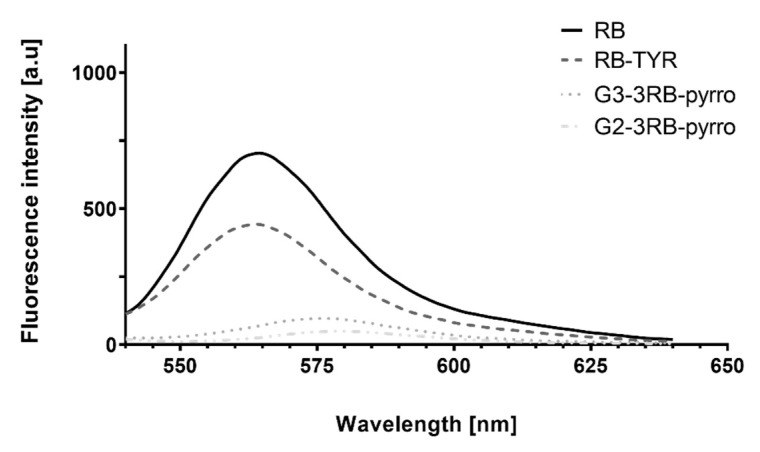
Fluorescence of rose bengal (RB), rose bengal modified with tyramine (RB–TYR) and conjugates of phosphorus dendrimers of the second and the third generation with three molecules of RB (G2–3RB–pyrro and G3–3RB–pyrro, respectively), dissolved in distilled water. Concentration of all compounds was calculated for RB and equaled 2.5 μM. Due to the lack of fluorescence activity of the G1–3RB–pyrro conjugate, data are not shown. Excitation wavelength: 525 nm.

Spectrofluorimetric analysis showed drastic changes of the spectral properties of RB upon modification with tyramine and subsequent conjugation to phosphorus dendrimers (Figure 6). The attachment of tyramine to RB caused a two-fold decrease in fluorescence intensity. Furthermore, the fluorescence of all conjugates was extremely low: G1–3RB–pyrro was non-fluorescent, the fluorescence of G2–3RB–pyrro and G3–3RB–pyrro was only slightly higher, and the fluorescence of G3 conjugate was sufficient for the next experiments. Moreover, the results showed the emission wavelength shift from 565 for RB and RB-TYR, to 575 and 580 nm for G3–3RB–pyrro and G2–3RB–pyrro, respectively.

### 2.3. Size and Zeta Potential Measurements

It was shown that the G3–3RB–pyrro conjugate retained the nanometric size and significant surface positive charge (Table 1), potentially favoring efficient endocytosis and cell entry [22]. However, when compared with our previous research [23], the size of the tested conjugate was enlarged, suggesting the possibility of aggregation.

### 2.4. Drug Release Studies

**Figure 7:**
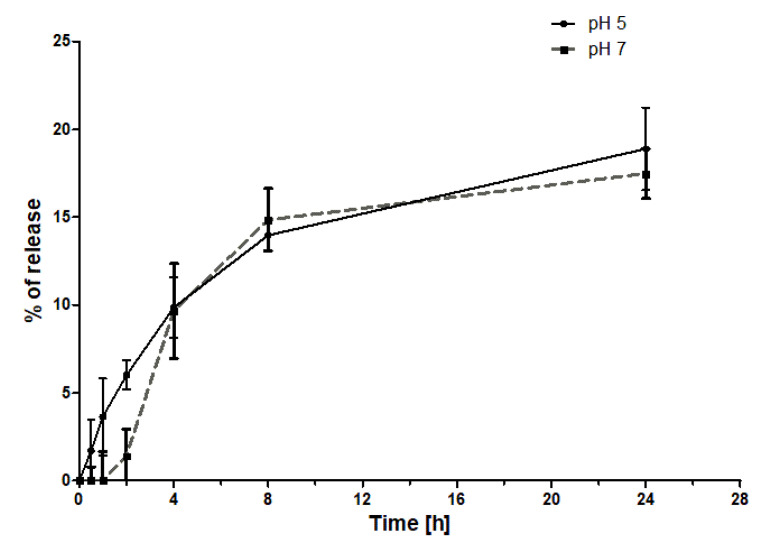
Release of RB from the G3–3RB–pyrro conjugate under different pH conditions. Data presented as mean ± SD.

The release of RB from the G3–3RB–pyrro conjugate was evaluated under two pH conditions (pH 5 and pH 7.4) (Figure 7). The results showed that in both cases, the release of RB is extremely low, plateauing after 8 h at 15% release. Furthermore, there was no significant difference between the release rates under both pH conditions.

### 2.5. Cellular Uptake Assay

**Figure 8:**
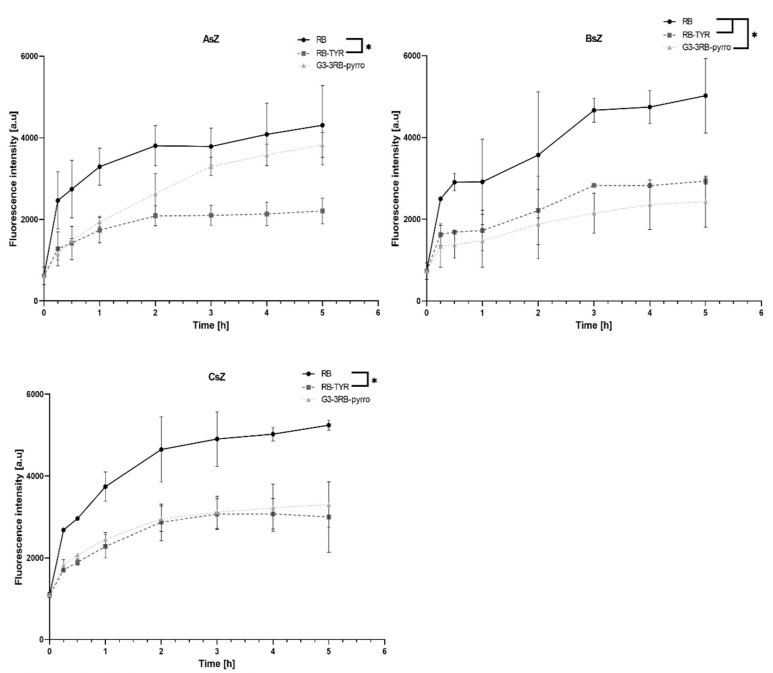
Cellular uptake of tested compounds (RB, RB–TYR, G3–3RB–pyrro at 5 µM final concentration of RB) by AsZ, BsZ and CsZ cell lines. Data presented as mean ± SD. * *p* < 0.05.

Uptake experiments showed significant differences in cell uptake rates between both compounds tested and cell lines (Figure 8). First, in all cell lines, RB showed similar potential to enter the cells, with its cellular uptake being the most efficient in comparison to other compounds. The modification of RB with tyramine decreased uptake level approximately by half, independently of cell line. Finally, the G3–3RB conjugate was characterized by moderate but slowed down cellular transport compared to free RB in the AsZ cell line, while for the BsZ and CsZ lines, cellular uptake was similar to RB–TYR.

### 2.6. Singlet Oxygen Generation

**Figure 9:**
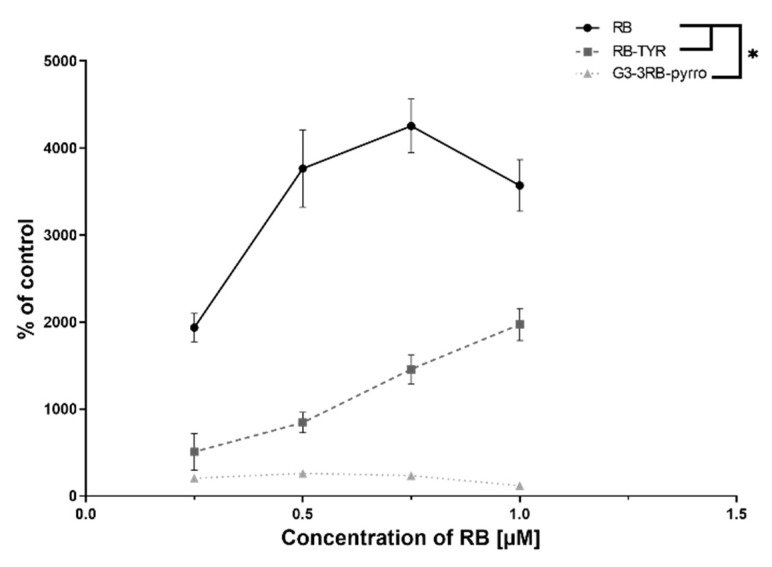
Comparison of singlet oxygen generation by free rose bengal (RB), rose bengal modified with tyramine (RB–TYR) and conjugate of phosphorus dendrimer of the third generation with rose bengal (G3–3RB–pyrro). Data presented as mean ± SD. * *p* < 0.05.

Singlet oxygen generation by the compounds under evaluation was assessed with the use of a highly selective Singlet Oxygen Green Sensor (SOSG) probe. It was found that the modification of RB with tyramine and subsequent attachment of such construct to the G3 dendrimer gradually reduced singlet oxygen production (Figure 9). The RB–tyramine conjugation caused a decrease in singlet oxygen generation in comparison with free RB, and the covalent binding of RB–TYR to the dendrimer further reduced the level of fluorescence.

### 2.7. Phototoxicity

**Figure 10:**
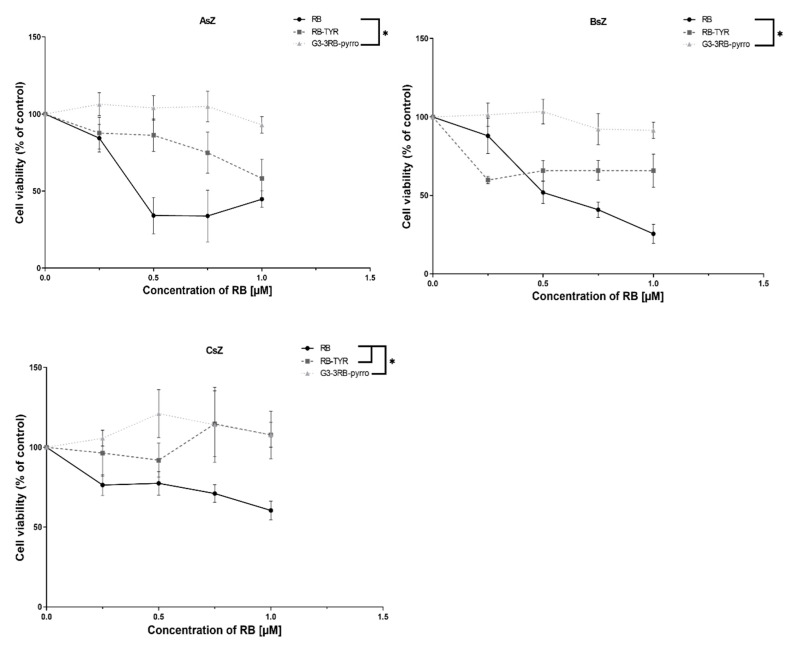
Phototoxic activity of rose bengal (RB), rose bengal modified with tyramine (RB–TYR) and conjugate of phosphorus dendrimer of the third generation with rose bengal (G3–3RB–pyrro). Data presented as mean ± SD. * *p* < 0.05.

Cytotoxicity studies performed with the use of MTT assay allowed to demonstrate an evident decrease in phototoxic activity with increasing modification of RB. In all cell lines, free RB showed the highest toxicity after irradiation, with RB–TYR being less toxic and G3–3RB–pyrro being almost non-toxic in the tested concentration range (Figure 10). Additionally, tested compounds were completely non-toxic without irradiation. 

## 3. Discussion

Photodynamic therapy is based on the application of a photosensitizer that, upon irradiation by light of a specified wavelength, produces reactive oxygen species and singlet oxygen [24], the generation of which starts a cascade of reactions ultimately leading to cell death [25]. The ideal photosensitizer is characterized by the following: maximum absorbance between 650 and 850 nm, high efficiency of free radical production, fluorescence emission, low photodegradation, non-toxicity in the dark, lack of allergic reactions and other side effects [26]. Unfortunately, despite many years of research on photosensitizers, so far, no drug has been developed that would be characterized by all of these features. Therefore, photosensitizers are being subjected to chemical modifications, e.g., being combined with nanoparticles. Major benefits of using nanoparticles in PDT include protection of the drugs from degradation, improvement of their solubility, prolongation of blood half-life, modulation of pharmacokinetic properties and targeted delivery [11,27]. Modern techniques of nanotechnology allow to synthesize numerous nanoparticles with various shapes, sizes and properties. Among them, dendrimers deserve special attention. These highly branched, monodisperse, polyvalent polymers of well-defined, regular structure are characterized by high solubility, biopermeability, and high loading capacity. Dendrimers, due to their shape, structure and nanometric size, may also efficiently penetrate into the tumor environment and retain in tumor interstitium, which is described as enhanced permeability and retention (EPR) effect [28]. Due to their three-dimensional architecture, they provide the possibility of covalent conjugation of the drug to the functional surface groups or encapsulation inside the dendritic structure [29]. 

In order to create covalent bonding between the drug and the dendrimers, the choice of appropriate linker is crucial. Linkers are molecules possessing two functional sides that can be used for joining chemical compounds together. Capability to design different kinds of linkers enable their application in drug development, proteomics, imaging and DNA sequencing. Nowadays it is possible to manufacture linker with strictly defined chemical composition and properties, including high stability in changeable environment or, on the other hand, the possibility of specific cleavage. By using different linkers, it is possible to release the therapeutic compound in a specified place and time, which can contribute to the reduction of side effects of classical drug application. This includes linkers that are sensitive to the change of pH or temperature, enzymatic or redox reactions, light irradiation, and hypoxia [30,31]. However, it has been shown that in case of PDT, the drug does not have to be released due to the specificity of its anticancer activity [11,32]. 

Previously, non-covalent complexes based on a phosphorus dendrimer and RB have been evaluated in vitro. The formation of non-covalent complexes [19,33] and their promising application in PDT [20] have been described. In the present paper, we focused on the evaluation of the ability of phosphorous dendrimers of different generations covalently modified with RB to act as efficient carriers for this drug. Thus, we used tyramine as a stable linker with suitable length to ensure proper exposition of RB on the surface of the dendrimer [34]. The presence of the phenol group allows for the formation of stable O-P bonds in the framework of the phosphorus dendrimer, as demonstrated previously [35]. Our main goal involved the increase in the stability of the delivery system, which may be vital in the case of in vivo experiments in comparison to non-covalent complexes. In the initial in vitro studies, we examined the properties and stability of conjugates of phosphorus dendrimers with RB–TYR, as well as their ability to generate singlet oxygen and exert phototoxic effect in vitro.

Three conjugates of phosphorus dendrimers of different generations with RB (G1–, 2–, 3–3RB–pyrro) were successfully synthesized and characterized. Interestingly, the spectrofluorimetric studies revealed that even a small chemical modification of RB by the attachment of tyramine reduces its fluorescence two-fold in comparison to free RB. The conjugation of RB to phosphorus dendrimers using a tyramine linker caused further reduction of fluorescence. This effect is not unexpected and was previously described by Li et al. [36]. In general, we observed a decrease in fluorescence intensity with a decreasing generation of phosphorus dendrimers to such an extent that the G1 dendrimer did not show any fluorescence. Thus, in the next experiments we used RB, RB–TYR as an intermediate form, and G3–3RB–pyrro as the conjugate with the highest fluorescence, which is important for effective PDT [37]. This conjugate retained its nano-size and positive surface electrostatic potential, which is important for efficient cellular uptake [22]. Furthermore, G3–3RB–pyrro was stable both in pH conditions of the bloodstream (pH 7.4) and the tumor environment (pH 5)—only a small amount of the drug was released from the conjugate, even after 24 h.

Since RB is a hydrophilic compound, it cannot penetrate the cell membrane by passive diffusion and most probably require specific membrane transporters. Repeated administration of the drug may lead to down-regulated of expression of those transporters, subsequently leading to drug resistance [38]. Therefore, we evaluated the cellular uptake of the tested compounds in order to check if the conjugation can improve intracellular delivery of the photosensitizer. First, we observed that the uptake level of RB–TYR was decreased approximately two-fold in comparison with free RB in all tested cell lines. This result may be caused by a reduced affinity of the chemically modified drug to membrane protein transporters. However, the conjugate in the AsZ cell line was uptaken as efficiently as the free drug, which indicated that the tested phosphorus dendrimer could act as an efficient nanocarrier. On the other hand, the uptake in the BsZ and CsZ cell lines was decreased to the level of RB–TYR. This phenomenon can be caused by differences in endocytosis processes in different cell lines, which was previously shown, e.g., for poly(propyleneimine) (PPI) glycodendrimers [39,40]. 

Since one of the most important feature of a photosensitizer is the generation of singlet oxygen, which is the basic mechanism responsible for death of cancer cells [41,42], we evaluated this process under the influence of the tested compounds. However, the results showed that RB–TYR exhibits decreased production of singlet oxygen in comparison to free RB, while G3–3RB–pyrro practically does not show this ability. These observations were consistent with the results of the analysis of fluorescence properties of the tested compounds and, moreover, they translated into the results of cytotoxicity tests, which showed that free RB has the highest phototoxicity, and with its progressive modification (attachment of tyramine and then the G3 dendrimer), the reduction of toxic effect occurs.

## 4. Materials and Methods

### 4.1. Materials

**Figure 11:**
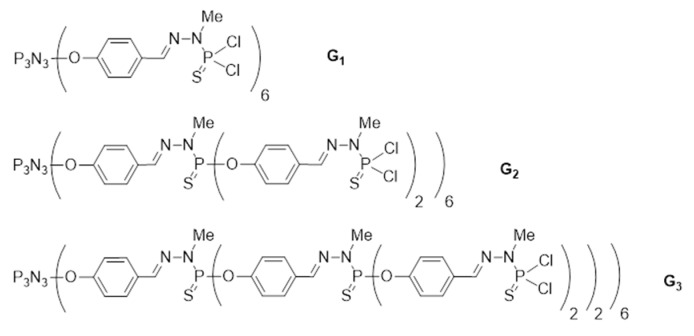
The schemes of G1, G2, G3 phosphorus dendrimers.

Phosphorus dendrimers (and their conjugates with RB) of the first, second and third generation were synthesized in the Laboratoire de Chimie de Coordination, CNRS (Figure 11).

Rose bengal, tyramine, cesium carbonate, thionyl chloride, 1-(2-aminoethyl)pyrrolidine, triethylamine (TEA), N,N-diisopropylethylamine (DIPEA), fetal bovine serum, penicillin/streptomycin solution, trypsin–EDTA solution, and MTT (3-(4,5-dimethyl-2-thiazolyl)-2,5-diphenyl-2H-tetrazolium bromide) were purchased from Sigma-Aldrich, Taufkirchen, Germany and TCI, Zwijndrecht, Belgium. Dulbecco’s phosphate buffered saline with no calcium and no magnesium (DPBS) was purchased from Biowest, France. 154 CF culture medium was obtained from Gibco, Thermo Fisher Scientific, Waltham, MA, USA. Chelex 100 Resin was obtained from Bio-Rad. Singlet oxygen sensor green (SOSG) was purchased from Molecular Probes, ThermoFisher, Waltham, MA, USA. Dimethyl sulfoxide (DMSO) was purchased from POCH (Gliwice, Poland). Snake-Skin™ Dialysis Tubing, 3.5K MWCO, 22 mm was obtained from ThermoFisher, Waltham, MA, USA. Murine basal cell carcinoma lines (AsZ, BsZ, CsZ) were kindly provided by Dr. Ervin Epstein (Children’s Oakland Research Institute, Oakland, CA, USA). 

### 4.2. Methods

#### 4.2.1. Preparation of Drug–Dendrimer Conjugates—General Information

All reactions were carried out in organic solvents using standard high vacuum and dry-argon techniques. All purchased chemicals were used without further purification. All solvents were dried and purified with the MBraun SPS 800 solvent purification system before use. ^1^H, ^13^C, and ^31^P NMR spectra were recorded with DPX300 and AV400 spectrometers (Bruker, Billerica, MA, USA). All ^13^C NMR and ^31^P NMR spectra were generally recorded decoupled {^1^H}. The signal of the nondeuterated solvent served as an internal standard.

IR spectra were recorded with Perkin Elmer Frontier (Waltham, MA, USA) and MALDI-TOF spectra with MALDI TOF MICROMASS WATERS (Milford, MA, USA).

#### 4.2.2. Synthesis of the Rose Bengal Acid Chloride

Rose bengal disodium salt (2 g; 2 mmol) was dried, dissolved in 20 mL of chloroform and cooled to 0 °C. Thionyl chloride (10 mL) was added and the resulting solution was stirred for 2 h at 60 °C under reflux and then stirred overnight at room temperature (RT). The reaction was carried out in an argon atmosphere. After completion of the reaction (checked by TLC), the solvent was removed under vacuum line at 50 °C. Solid residue was characterized by IR with appearance of a band at 1780 cm^−1^ corresponding to acid chloride. Crude solid residue was carried forward as such for next step, because purification leads to the conversion of chloride into acid.

#### 4.2.3. Synthesis of Rose Bengal–Tyramine

**Figure 12:**
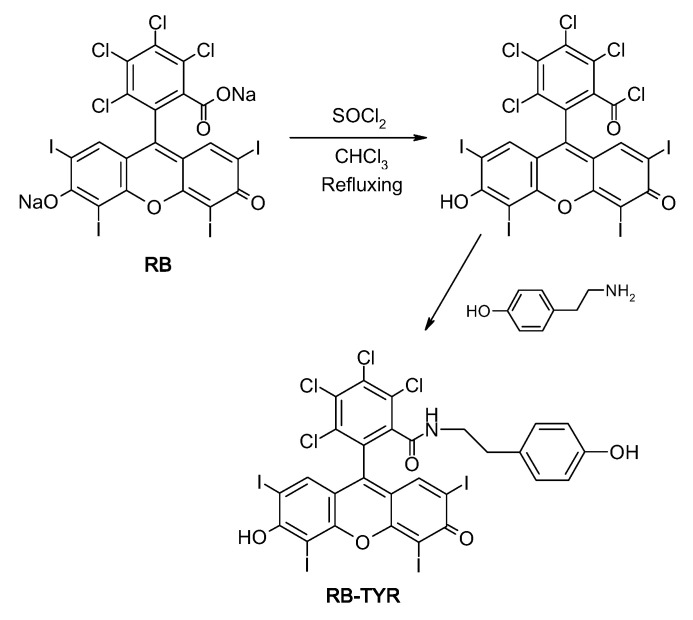
The scheme of the RB–TYR synthesis.

RB acid chloride (0.5 g, 0.5 mmol) was mixed with tyramine (0.1 g; 0.65 mmol), dried and kept in THF (30 mL). In the next step TEA (0.65 mmol) was added. The resulting mixture was stirred for 24 h. After completion of the reaction (checked by TLC), the solvent was evaporated under vacuum. Crude residue was purified on silica column using ethyl acetate and methanol as a mobile phase (20% of methanol and 80% of ethyl acetate). The scheme of the rose bengal modification is presented in Figure 12.

**Figure 13:**
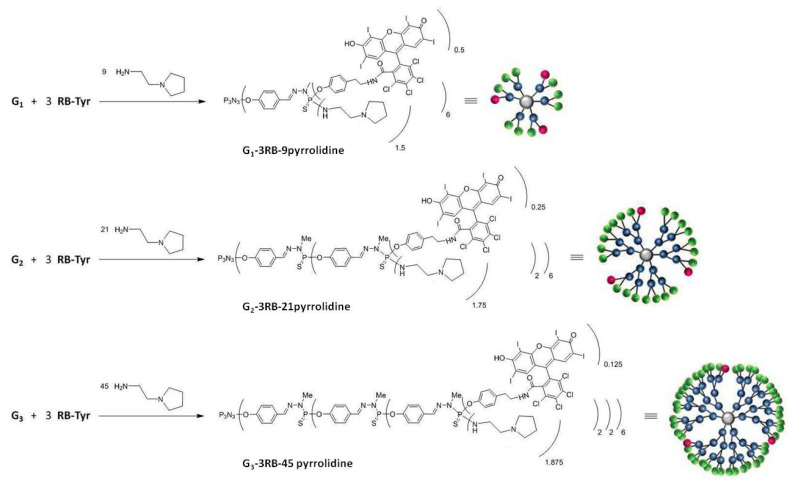
The scheme of the synthesis of the G1–, 2–, 3–RB–pyrrolidine conjugates.

The rose bengal modified with tyramine was characterized by NMR (^1^H and ^13^C NMR). ^1^H NMR (DMSO-d6; ppm): δ 9.36 (s, 1H, NHCO), 7.84 (s, 2H, OH), 7.32 (s, 2H, ArH_RB_), 7.00 (d, J = 8.4 Hz, ^2^H, CHAr_TYR_), 6.69 (d, J = 8.4 Hz,2H, CHAr_TYR_), 2.90 (t, J = 8.6 Hz, 2H, CH_2_NHCO), 2.70 (t, J = 8.6 Hz, 2H, CH2Ar_TYR_). ^13^C NMR (DMSO-d6; ppm): δ 172.09 (Ar_RB_=O, Ar_RB_-O^-^), 162.09 (NCO), 157.88 (Ar_RB_-O), 156.63 (Ar-C-Cl), 137.37 (Ar_RB_-Cl, C_TYR_-CH_2_), 130.91 (Ar_TYR_), 130.26 (Ar_RB_-Cl), 115.81 (Ar_TYR_), 111.61, 97.12 (Ar_RB_-I), 97.07 (Ar_RB_-I), 76.18 (C-CONH), 44.09 (CH_2TYR_), 34.01 (CH_2TYR_). IR spectra (neat) show the disappearance of the band at 1780 cm^−1^ and the appearance of a new band at 1614 cm^−1^, which confirms the conversion of acid chloride into the corresponding phenol. Mass spectra corroborate the formation of the desired RB–tyramine derivative (Figure 13). 

#### 4.2.4. Synthesis of Rose Bengal–G1, G2, G3 Phosphorus Dendrimers—Pyrrolidine Conjugates

##### Preparation of the PS-Cl_2_ G1, G2, G3 Dendrimer Conjugates Containing Three Molecules of Rose Bengal

RB–TYR (65 µmol) and cesium carbonate (65 µmol) were dried under vacuum, dissolved in 20 mL of THF and kept in dark at RT. A total of 21 µmol of PS-Cl_2_ G1 or PS-Cl_2_ G2, or PS-Cl_2_ G3 dendrimer were added. The resulting mixtures were stirred for 72 h in an argon atmosphere. After completion of the reaction, the solvent was evaporated under reduced pressure. The residue was dissolved in 10 mL THF and precipitated in 100 mL pentane. 

The structure of the G1–3RB conjugate was controlled by ^31^P NMR, ^1^H NMR, ^13^C NMR in THF-d8. ^31^P NMR: δ 8.09 (s, P_0_), 61.99 (s, P_1_), 73.13 (s, P_1_-tyramine) ppm; ^1^H NMR: 7.60 (m, 18H, ArH_Den_ and CH=N), 7.24–6.99 (m, 24H, ArH_Den_, ArH_RB_, ArH_TYR_), 6.68 (d, J = 7.2 Hz, 6H, ArH_TYR_), 3.52–3.38 (m, 18H, P-N-CH_3_), 3.10 (s, 6H, CH_2_-NH), 2.94 (s, 6H, CH_2_-Ar_TYR_); ^13^C NMR: δ 162.37 (NCO), 158.39(C_Ar_O), 156.32 (Ar_TYR_-O), 152.34 (C_Ar-Den_), 139.44 (CH=N), 139,22 (C_RB_-Cl), 137.18 (C=C-I), 136.65 (C_RB_-CO), 135.81 (Ar_TYR_), 131.04 (C_Ar-Den_), 129.49 (Ar_TYR_), 129.06 (C_RB_-Cl), 129.03 (C_Ar-Den_), 128.11 (C_RB_-Cl), 127.74 (C_RB_-Cl), 124.53(C_Ar-RB_), 120.94 (C_Ar-Den_), 115.16 (Ar_TYR_), 94,49 (C_RB_-I), 78,20 (C_RB_), 44.09 (CH2_TYR_), 34.01 (CH2_TYR_), 29.72(br.s., P-N-CH_3_). 

The structure of the G2–3RB conjugate was controlled by ^31^P NMR, ^1^H NMR, ^13^C NMR in THF-d8. ^31^P NMR: δ 8.39 (s, P_0_), 62.24 (s, P_1_), 62.40 (s, P_2_), 73.26 (s, P_2_-tyramine) ppm; ^1^H NMR: δ 7.76–7.55 (m, 54H, ArH_Den_ and CH=N), 7.27 (d, J = 16.8, d, J =8.8 Hz, 36H, ArH_Den_), 7.09–7.06 (m, 6H, ArH_TYR_), 7.02–7.00 (m, 6H, ArH_RB_), 6.68 (d, J = 2.6 Hz, 6H, ArH_TYR_), 3.46 (d, J = 14.0 Hz, 36H, P-N-CH_3_), 3.34 (d, J = 10.4 Hz, 18H, P-N-CH_3_), 2.88–2.63 (m, 12H, CH_2_-NH, CH_2_-Ar); ^13^C NMR: δ 162.42 (NCO), 158.39(C_Ar_O), 156.27 (Ar_TYR_), 151.92 (C_Ar-Den_), 151.36 (C_Ar-Den_), 148.13 (C_RB_-Cl), 141.88 (CH=N), 139.48 (CH=N), 136.66(C_RB_-Cl), 135.86(Ar_TYR_), 132.38 (C_RB_-Cl), 131.85 (C_Ar-Den_), 131.07 (C_Ar-Den_), 129.55(Ar_TYR_), 128.51 (C_RB_-Cl), 128.10(C_Ar-Den_), 127.76 (C_Ar-Den_), 124.90 (C_RB_-Cl), 123.93 (C_Ar-RB_), 121.64(C_Ar-Den_), 121.10(C_Ar-Den_), 115.19 (C_ArRB_-OH), 78.82 (C_RB_-I), 78.26 (C_RB_), 44.00 (CH_2TYR_), 34.11 (CH_2TYR_), 31.30 (br.s.P-N-CH_3_).

The structure of the G3–3RB conjugate was controlled by ^31^P NMR, ^1^H NMR, ^13^C NMR in CDCl_3_. ^31^P NMR: δ 8.30 (s, P_0_), 61.90 (s, P_2_), 62.24 (s, P_1_), 62.78 (s, P_3_), 72.55 (s, P_3_-tyramine) ppm; ^1^H NMR: δ 7.97–7.35 (m, 116H, ArH_Den_ and CH=N), 7.21 (m, 72H, ArH_Den_), 7.02–6.90 (m, 6H, ArH_Tyr_), 6.90 (s, 6H, ArH_RB_), 6.70 (d, J = 6.8 Hz, 6H, ArH_TYR_), 3.46–3.18 (m, 126H, P-N-CH_3_), 2.84 (d, J = 11.4 Hz, 6H, CH_2_-NH, CH_2_-Ar), 2.72(s, 6H, CH_2_-Ar_TYR_); ^13^C NMR: δ 156.81 (NCO), 155.22 (C_Ar_O), 151,79 (Ar_TYR_), 151.79 (Ar-CCl), 151.73 (C_Ar-Den_), 151.35 (C_Ar_-_Den_), 140.86 (C_RB_-Cl), 140,68 (CH=N), 139.10 (CH=N), 136.24 (Ar_TYR_), 132.21 (C_RB_-Cl), 131.52 (C_Ar_-_Den_), 130.05 (C_Ar_-_Den_), 129.42 (Ar_TYR_), 128.75 (C_Ar_-_Den_, C_RB_-Cl), 128.35 (C_Ar_-_Den_), 121.81 (C_Ar_-_Den_), 121.28 (C_Ar_-_Den_, Ar_RB_), 115.72 (CAr_RB_-OH), 77.33 (C_RB_-I), 74.08 (C_RB_-I), 68.57 (C_RB_), 42.84 (CH_2TYR_), 33.14 (CH_2TYR_), 30.62 (br.s. P-N-CH_3_). 

##### Preparation of the PS-Cl_2_ G1, G2, G3 Dendrimer Conjugates Containing Three Molecules of Rose Bengal and 9, 21 and 45 Molecules of Pyrrolidine, Respectively

**Figure 14:**
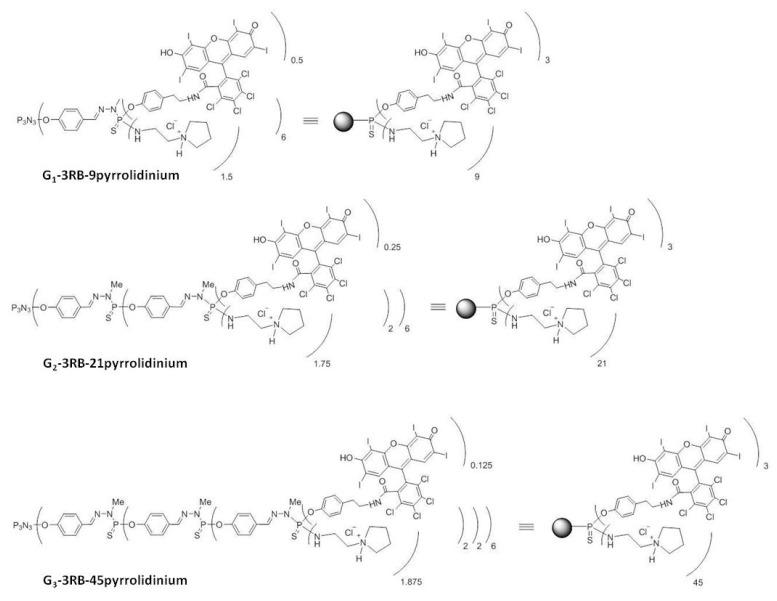
The structures of the G1–, 2–, 3–RB–pyrrolidinium conjugates. Black spheres are graphical representation of non-modified phosphorus dendrimer.

The conjugates (16 µmol) bearing 3 RB units on their surface and 9, 21 or 45 terminal P-Cl bonds in 20 mL of THF were mixed with the excess of anhydrous sodium sulphate. The solution was cooled to 0 °C. (DIPEA) (144, 336 and 730 µmol, respectively) was added, followed by the slow addition of 1-(2-aminoethyl)pyrrolidine (144, 336 and 730 µmol, respectively), and then the solution was stirred for 6h. After completion of the reaction controlled by ^31^P NMR, the solvent was evaporated under reduced pressure. The residue was extracted with dichloromethane (DCM) (20 mL) and 10% K_2_CO_3_ in water (10 mL). The organic layer was dried over magnesium. The residue was again dissolved in 10 mL THF and precipitated in 100 mL pentane. The last step involved the protonation of the grafted amino groups with HCl, subsequently leading to the formation of charged phosphorus dendrimers G1–3RB–9 pyrrolidinium, G2–3RB–21 pyrrolidinium and G3–3RB–45 pyrrolidinium (Figure 14).

The structure of the G1–RB–pyrrolidine conjugate was controlled by ^31^P NMR, ^1^H NMR, ^13^C NMR in CDCl_3_. ^31^P NMR: δ 8.07 (s, P_0_), 68.45 (s, P_1_-RB), 68.80 ppm (s, P_1_-pyrrolidine); ^1^H NMR: δ 7.46 (d, J = 10.6 Hz, 18H, ArH_Den_ and CH=N), 7.18–6.50 (m, 30H, ArH_Den_, ArH_RB_, ArH_TYR_), 3.15 (d, J = 9.4 Hz, 18H, P-N-CH_3_), 3.07–2.97 (m, 30H, CH_2_-Ar, CH_2_NH), 2.58–2.52 (m, 54H, CH_2_N, NCH_2_), 1.79–1.76 (m, 36H, CH_2_); ^13^C NMR of G1–RB–pyrrolidine: δ 158.19(C_Ar_O), 156.80 (NCO) 156.32 (Ar_TYR_), 152.34 (C_Ar-Den_), 151.79 (Ar-CCl), 139.44 (CH=N) 139,22 (C-Cl), 137.18 C-I), 136.60 (C_RB_-CO), 135.75 (Ar_TYR_),131.04 (C_Ar-Den_), 129.49 (Ar_TYR_), 129.06 (C_RB_-Cl), 129.03 (C_Ar-Den_), 128.11 (C-Cl), 127.74 (C_Ar-Den_), 127.51 (Ar_TYR_, Ar_RB_), 121.12 (Ar_TYR_), 121.05 (C_Ar-Den_), 84.88 (C_RB_-I), 56.44 (CH_2_), 39,81 (N-CH_2_), 39.81 (CH_2_-N), 37.07 (CH_2TYR_-NH), 36.56 (CH_2TYR_), 31.91 (P-N-CH_3_), 25.59 (CH_2_). 

The structure of the G2–RB–pyrrolidine conjugate was controlled by ^31^P NMR, ^1^H NMR, ^13^C NMR in CDCl_3_. ^31^P NMR: δ 8.48 (s, P_0_), 62.89 (s, P_1_), 68.76 (s, P_2_-RB), 69.40 (s, P_2_-pyrrolidine) ppm; ^1^H NMR: δ (m, 54H, ArH_Den_ and CH=N), 7.18 (s, 36H, ArH_Den_), 7.10 (d, J = 6.8 Hz, 6H, ArH_TYR_), 7.04 (s, 6H, ArH_RB_), 6.7 (d, J = 6.8 Hz, 6H, ArH_TYR_), 3.40–3.27 (m, 36H, P-N-CH_3_), 3.26 (d, J = 5.8 Hz, 18H, P-N-CH_3_), 3.19 (d, J = 6.2 Hz, 6H, CH_2_-NHPS), 3.15–3.07 (m, 48H, CH_2_NHCO, CH_2_-Ar_TYR_), 2.53–2.48 (m, 126H, CH_2_N, NCH_2_); ^13^C NMR: δ 171.96 (NCO), 164.85 (C_Ar_O), 137.21 (C_Ar-Den_, C_RB_-Cl), 129.16 (C_Ar-Den_, CH_TYR_), 127.80 (C_Ar-Den_), 127.15 (C_Ar-Den_, C_RB_-Cl), 124.86 (C_Ar-Den_), 121.30 (C_Ar-Den_), 115.03 (Ar-C-OH), 84.18 (C_RB_-I), 53.67 (CH_2_-NH_2_), 39.75 (CH_2TYR_), 39.73 (CH_2_-N, N-CH_2_), 39.04 (CH_2TYR_), 32.60 (P-N-CH_3_), 29.72 (P-N-CH_3_), 26.96 (CH_2_).

The structure of the G3–RB–pyrrolidine conjugate was controlled by ^31^P NMR, ^1^H NMR, ^13^C NMR in CDCl_3_. ^31^P NMR: δ 8.02 (s, P_0_), 61.69 (s, P_2_), 62.07 (s, P_1_), 67.69 (s, P_3_-RB), 68.38 (s, P_3_-pyrrolidine) ppm; ^1^H NMR: δ 7.69–7.40 (m, 116H, ArH_Den_ and CH=N) 7.25–6.51 (m, 102H, ArH_Den_, ArH_RB_, ArH_TYR_), 3.17-3.02 (m, 90H, CH_2_-NHPS), 3.14–3.00 (m, 126H, P_1_-N-CH_3_), 2.67–2.52 (m, 12H, CH_2_-NH, CH_2_-Ar_TYR_), 2.50–2.41 (m, 270H, CH_2_-N, N-CH_2_), 1.89–1.69 (m, 180H, CH_2_); ^13^C NMR: δ 156.80 (NCO), 156.32 (Ar_TYR_), 152.34 (C_Ar-Den_), 151.69 (Ar-CCl), 136.60 (C_RB_-CO, 135.40 (CH=N) 133,12 (C-Cl), 132.18 C-I), 135.75 (Ar_TYR_),131.04 (C_Ar-Den_), 129.49 (Ar_TYR_), 129.06 (C_RB_-Cl), 129.03 (C_Ar-Den_), 128.11 (C-Cl), 127.74 (C_Ar-Den_,), 127.51 (Ar_TYR_, Ar_RB_), 121.12 (Ar_TYR_), 121.05 (C_Ar-Den_), 84.88 (C_RB_-I), 56.44 (CH_2_), 39,81 (N-CH_2_), 39.81 (CH_2_-N), 37.07 (CH_2TYR_-NH), 36.56 (CH_2TYR_), 30.98 (P-N-CH_3_), 23.58 (CH_2_). 

#### 4.2.5. Spectroscopy Studies

Fluorescence spectra were acquired using a PerkinElmer LS-50B spectrofluorometer (Waltham, MA, USA). All measurements were performed in distilled water, at RT. The excitation wavelength was set to 525 nm and spectra were collected in a wavelength range from 540 nm to 640 nm. Excitation and emission slits were 5 nm and 7 nm, respectively. The measurements were carried out for tested compounds: RB, RB-TYR, and G1–, 2–, 3–RB–pyrrolidinium (G1–, 2–, 3–3RB–pyrro) conjugates (Figure 14) at 2.5 μM concentration of RB. 

#### 4.2.6. Size and Zeta Potential Measurements

Measurements of size and zeta potential were performed with the use of Zetasizer Nano ZS (Malvern Instruments Ltd., Malvern, UK). Water solutions containing the studied compound at the final dendrimer concentration of 10 µM were placed in the low volume sizing cuvettes (ZEN0112, Malvern) for size determination or in the folded capillary cells (DTS 1070, Malvern) for zeta potential measurements and measured at 25 °C. The data were analyzed using the Malvern software.

#### 4.2.7. Drug Release Studies

In order to evaluate the rate of photosensitizer release from the conjugate (G3–3RB–pyrro) under different pH conditions, a 50 µM solution of G3–3RB–pyrro in DPBS was enclosed in a dialysis membrane tubing (SnakeSkin™ Dialysis Tubing, 3.5K MWCO, 22 mm, ThermoFisher, Waltham, MA, USA) and immersed in DPBS at a pH of 5 or 7.4 at RT. Samples from the internal phase were collected at subsequent intervals (0.5, 1, 2, 4, 8, 24 h) and the RB-TYR fluorescence similarly to spectroscopy studies. Percentage of release was determined with regards to the first sample, where the drug was not released from the conjugate (100% of initial fluorescence). The experiment was performed three times.

#### 4.2.8. Cell Culture

Murine basal cell carcinoma lines were cultured in 154-CF medium with 5% penicillin/streptomycin, 0.05 mM calcium, and 2% chelexed, heat-inactivated fetal bovine serum (FBS). Cells were cultured in T-75 culture flasks at 37 °C, 5% CO_2_ and subcultured every 2 or 3 days. The number of viable cells was determined by trypan blue exclusion assay with the use of Countess Automated Cell Counter (Invitrogen, Carlsbad, CA, USA). For the harvesting of the cells, a 0.25% (*w*/*v*) trypsin−0.03% (*w*/*v*) EDTA solution was used.

#### 4.2.9. Cellular Uptake Determination

To define cellular uptake of tested compounds, AsZ, BsZ and CsZ cells were seeded into 12-well plates at a density of 2 × 10^5^ cells per well. After 24 h incubation (37 °C, 5% CO_2_) cells were treated with selected compounds (RB, RB–TYR, G1–3–3RB–pyrro) at 5 µM of the final concentration of RB up to 5 h. Following the incubation, cells were collected using the trypsin–EDTA solution, washed with DPBS and suspended in fresh medium. To estimate the cellular uptake, fluorescence of the samples was measured using flow cytometer (LSRII, Becton Dickinson, BD Biosciences, city, if any state, San Jose, CA, USA). The excitation and the emission filters were 520 and 570 nm, respectively. 

#### 4.2.10. Singlet Oxygen Generation Assay

To determine singlet oxygen production, Singlet Oxygen Sensor Green (SOSG) probe at 1 μM concentration was used. The solutions of RB, RB–TYR and G3–3RB–pyrro were prepared in DPBS. The samples were prepared at 0.25, 0.5, 0.75 and 1 μM concentration of RB. Upon sample preparation, 100 μL of each solution was transferred to the 96-well black plate. All measurements were recorded using fluorescence microplate reader (Fluoroscan Ascent FL, ThermoFisher, Waltham, MA, USA). The excitation wavelength was 500 nm, and the emission wavelength was 538 nm. Samples were shaken before every measurement. The first measurement was recorded with no SOSG probe to determine whether RB, RB–TYR or G3–3RB–pyrro emit fluorescence in this range. Following the first measurement, SOSG was added to each well, and the fluorescence of the probe without irradiation was checked. Next, the plate was immediately placed under the Q.Light Pro Unit lamp equipped with a filter emitting visible light in the range of 385−780 nm (Q.Light, Rorschach, Switzerland). The plate was irradiated, and the fluorescence of the probe was measured in the course of irradiation time (5–60 min). Then, the slopes of the fluorescence curves were considered for the measurement of singlet oxygen generation. The results were presented as percentage of singlet oxygen generation in control (PBS).

#### 4.2.11. Cytotoxicity Studies

To measure the cytotoxicity, AsZ, BsZ and CsZ cells were seeded into 96-well plates at a density of 3 × 10^4^ cells per well. After 24 h incubation, the culture medium was aspirated from the wells. Then, 100 μL of the free photosensitizer (RB, RB–TYR or G3–3RB–pyrro) was added to the cells at final RB concentrations of 0.25, 0.5, 0.75 and 1 μM in fresh culture medium. Cells were incubated with tested compounds for 5 h (37 °C, 5% CO_2_). Then, the medium was replaced with fresh DPBS buffer, and cells were irradiated with visible light using a lamp (Q.Light Pro Unit Q.Light, Rorschach, Switzerland). The time of irradiation was 30 min. Immediately after irradiation, DPBS was replaced with fresh culture medium, and cells were incubated for 24 h as post PDT incubation. Additionally, the “dark” toxicity (without irradiation) was evaluated. Then, cell viability was measured using MTT assay. MTT was added to the wells at a concentration of 0.5 mg/mL and the plates were incubated for 3 h (37 °C, 5% CO_2_). After incubation, formazan crystals were dissolved in DMSO and the absorbance was read at 570 nm using the PowerWave HT Microplate Spectrophotometer (BioTek, Winooski, VT, USA)

#### 4.2.12. Statistical Analysis

For statistical significance testing, one-way ANOVA for concentration series and post hoc Tukey’s test for pairwise difference testing were used. In all tests, *p*-values < 0.05 were considered to be statistically significant. Data are presented as arithmetic mean ± SD.

## 5. Conclusions

The present work demonstrates that it was possible to (1) modify the structure of rose bengal—a well-known photosensitizer used for photodynamic therapy studies—with a linker in the form of tyramine; (2) to graft selectively three modified rose bengal units onto the surface of phosphorus dendrimers of generation 1, 2 and 3 used as nanocarriers; (3) to modify these new nanoobjects via the grafting with pyrrolidino groups, followed by their protonation; (4) to study in depth their physicochemical and biological properties; (5) to point out the key role of the linker between phosphorus dendrimers and rose bengal in limiting fluorescence and phototoxic properties of the photosensitizer.

Investigations are underway with the aim of preparing similar conjugates in order to examine the impact of the nature of the linker, the generation of the dendrimers, the nature of the amino groups, and the number of grafted rose bengal units (or other photosensitizers) on the in vitro and in vivo properties of these macromolecules.

Dendrimers are chemically well-designed drug nanocarriers, and the application of an appropriate linker allows to prepare stable compounds for in vitro tests. However, we observed a weakened photodynamic effect of RB upon conjugation of the drug to phosphorus dendrimers via the tyramine linker, which excludes this type of linker from further studies on dendrimer-based carriers for this photosensitizer. The most promising strategy in this case would be the selection of a linker that ensures stability in systemic circulation and specific release of the unmodified drug inside the tumor cells, thus ensuring its unchanged structure and phototoxic properties. In subsequent studies, we plan to screen different linkers and types of drug–dendrimer bonding in order to choose the best solution for the preparation of a RB–phosphorus dendrimer conjugate.

## Figures and Tables

**Table 1:** Size and zeta potential of the G3–3RB–pyrro conjugate. Data presented as mean ± SD.

	Hydrodynamic Diameter (nm)	Zeta Potential (mV)
G3–3RB	18.17 ± 4.96	44.54 ± 2.42
